# Slight Changes in the Gut Microbiome in Early-stage Chronic Kidney Disease of Unknown Etiology

**DOI:** 10.1264/jsme2.ME22097

**Published:** 2023-08-25

**Authors:** Ditsayathan Banjong, Thatsanapong Pongking, Na T. D. Tran, Somchai Pinlaor, Rungtiwa Dangtakot, Kitti Intuyod, Sirirat Anutrakulchai, Ubon Cha’on, Porntip Pinlaor

**Affiliations:** 1 Biomedical Science Program, Graduate School, Khon Kaen University, Khon Kaen, Thailand; 2 Faculty of Medical Laboratory Science, Danang University of Medical Technology and Pharmacy, Danang, Vietnam; 3 Department of Parasitology, Faculty of Medicine, Khon Kaen University, Khon Kaen, Thailand; 4 Faculty of Medical Technology, Nakhonratchasima College, Nakhon Ratchasima, Thailand; 5 Department of Pathology, Faculty of Medicine, Khon Kaen University, Khon Kaen, Thailand; 6 Department of Medicine, Faculty of Medicine, Khon Kaen University, Khon Kaen, Thailand; 7 Department of Biochemistry, Faculty of Medicine, Khon Kaen University, Khon Kaen, Thailand; 8 Center for Research and Development of Medical Diagnostic Laboratories, Faculty of Associated Medical Sciences, Khon Kaen University, Khon Kaen, Thailand; 9 Chronic Kidney Disease Prevention in Northeastern Thailand, Khon Kaen, Thailand

**Keywords:** gut microbiome, chronic kidney disease, 16S rRNA, next-generation sequencing, short-chain fatty acids

## Abstract

Gut dysbiosis and changes in short-chain fatty acids (SCFAs) occur in end-stage chronic kidney disease (CKD); however, the degree of these changes in the gut microbiome and serum SCFA profiles in the early stages of CKD,‍ ‍particularly in‍ ‍CKD‍ ‍of unknown etiology (CKDu), is unclear. We herein investigated the gut microbiome and SCFA profiles of early-stage CKD patients (CKD stages 1–3) in a community in Khon Kaen Province, Thailand. Seventy-two parasite-free participants were distributed among a healthy control group (HC, *n*=18) and three patient groups (an underlying disease group [UD, *n*=18], early-stage CKD with underlying disease [CKD-UD, *n*=18], and early-stage CKD of unknown etiology, [CKDu, *n*=18]). Fecal DNA was individually extracted and pooled for groups of six individuals (three pools in each group) to examine the composition of the gut microbiome using next-generation sequencing. A SCFA ana­lysis was performed on serum samples from each individual using gas chromatography-mass spectrometry. The results revealed that microbial abundance differed between the healthy group and all patient groups (UD, CKD-UD, and CKDu). *[Eubacterium]_coprostanoligenes_*group was more abundant in the CKDu group than in the HC and CKD-UD groups. Furthermore, serum concentrations of acetate, a major SCFA component, were significantly lower in all patient groups than in the HC group. The present results indicate that minor changes in the gut microbiome and a significant decrease in serum acetate concentrations occur in early-stage CKDu, which may be important for the development of prevention strategies for CKD patients.

The human gastrointestinal tract harbors a range of organisms that include bacteria, fungi, viruses, archaeans, and parasites (protozoa and helminths). Collectively, this assemblage is known as the gut microbiome and consists largely of bacteria (Vemuri *et al.*, 2020). The abundance and diversity of the microbiome depends on a number of factors *e.g.* age, sex, host genetics, the host immune system, an individual’s diet, and the use of antibiotics (Jandhyala *et al.*, 2015). Commensal bacteria typically dominate in the intestinal tract and, hence, have a significant impact on the health and physiology of the host, partly by excluding the establishment of pathogenic microorganisms and maintaining intestinal homeostasis and immune regulation. Furthermore, they help the host to degrade dietary fiber and complex carbohydrates into short-chain fatty acids (SCFAs) (Magliocca *et al.*, 2022; Portincasa *et al.*, 2022; Yao *et al.*, 2022). Therefore, gut dysbiosis disturbs host metabolism and has been implicated in the pathogenesis of several diseases, including inflammatory bowel disease, diabetes, obesity, cardiovascular diseases, hypertension, and chronic kidney disease (CKD) (Hills *et al.*, 2019; Wehedy *et al.*, 2021; Huang *et al.*, 2022).

CKD is a condition that is characterized by a gradual loss of kidney function with obvious implications for health (Neuen *et al.*, 2017). CKD is the most common form of kidney disease, with an estimated prevalence worldwide of up to 10% (GBD 2013 Mortality and Causes of Death Collaborators). The prevalence of CKD in northeastern Thailand was previously reported to be 22.2%, which is markedly higher than that in the central and southern regions of Thailand (Ingsathit *et al.*, 2010). Metabolic diseases, including diabetes, hypertension, and obesity, are important underlying diseases and have been identified as risk factors for the development of CKD and its progression to end-stage renal disease (ESRD) (Kazancioǧlu, 2013).

In addition to the high prevalence of CKD (Cha’on *et al.*, 2020), this region also has a high prevalence of parasitic infections, most commonly due to *Opisthorchis viverini* and *Strongyloides stercoralis* (Boonjaraspinyo *et al.*, 2013). Recent findings demonstrated that infections with *O. viverrini* in hamsters (Haonon *et al.*, 2021) and with *S. stercoralis* in otherwise healthy subjects (Nguyen *et al.*, 2022) and in CKD patients (Hai *et al.*, 2022) induced changes in the gut microbiome. In a previous study in the same community, *S. stercoralis* infection was found to induce gut dysbiosis and was associated with progression of CKD (Hai *et al.*, 2022).

The prevalence of CKD of unknown etiology (CKDu) is low in Thailand (<1%) (Aekplakorn *et al.*, 2021), whereas that in Khon Kaen Province, northeastern Thailand, is high (approximately 28.9% of CKD patients or 7.76% of the total screened population) (Cha’on *et al.*, 2020). Our research group is currently investigating whether this high prevalence of CKDu is a consequence of environmental pollution, as suggested by Cha’on *et al.* (2020). The present study provides baseline information on the microbiome of CKDu patients in northeastern Thailand as a context for future work.

Changes in the gut microbiome have been associated with the progression of CKD (Kim and Song, 2020; Wehedy *et al.*, 2021). The 16s rRNA gene-sequencing approach revealed that the abundance of *Enterococci* and *Enterobacteria* species increased in CKD patients (Vaziri *et al.*, 2013). Moreover, the abundance of pathogenic bacteria was significantly increased in patients with ESRD, with a corresponding decrease in that of beneficial bacteria, such as *Bifidobacteria*, *Prevotellaceae*, and *Bacteroidaceae* (Sampaio-Maia *et al.*, 2016). An imbalance between beneficial and pathogenic bacteria leads to gut dysbiosis, which contributes to an increase in toxic metabolites, such as those associated with the progression and complications of CKD (Huang *et al.*, 2022; Shivani *et al.*, 2022; Wang *et al.*, 2020). In addition, a reduction in SCFA-producing bacteria has been reported in ESRD (Wang *et al.*, 2019). SCFAs are fatty acids with fewer than six carbon atoms and are generated by the microbial fermentation of indigestible polysaccharides. SCFAs, such as acetate and butyrate, are important metabolites for maintaining intestinal homeostasis (Magliocca *et al.*, 2022; Yao *et al.*, 2022). These SCFAs act as a primary energy source for colonocytes and maintain the integrity of the intestinal barrier through their anti-inflammatory effects (Huang *et al.*, 2022). SCFAs are reduced in various diseases, particularly CKD (Lu *et al.*, 2021; Magliocca *et al.*, 2022). Therefore, gut microbiome changes and reduced SCFAs are associated with the development and progression of CKD (Chen *et al.*, 2019; Hobby *et al.*, 2019; Lu *et al.*, 2021).

Previous studies reported changes in the gut microbiome and serum concentrations of SCFAs in end-stage CKD patients (Jadoon *et al.*, 2018; Wong *et al.*, 2014). However, limited information is currently available on the concentrations of SCFAs and changes in the gut microbiome during the early stages of CKD (stages 1 to 3 and including CKDu) and in the presence of other underlying diseases, such as diabetes and hypertension. Therefore, the present study investigated the composition of the gut microbiome and serum concentrations of SCFAs in the early stages of CKD with a focus on CKDu in a rural community in Khon Kaen Province, Thailand. To reduce the possible confounding effect of intestinal parasites, only participants free of parasites (based on a stool examination) were included in the present study. Three groups of patients (early-stage CDK of unknown etiology [CKDu]; early-stage CKD with underlying diseases [CKD-UD]; and patients with underlying diseases, but not CKD [UD]) and one group of healthy controls (HC) were enrolled in this study. The results obtained herein will provide a more detailed understanding of the gut microbiota and SCFA concentrations in early-stage CKD, which may lead to better approaches for the diagnosis of early-stage CKD and therapeutic strategies to prevent its progression (Feng *et al.*, 2018; Alsharairi, 2022; Caetano and Castelucci, 2022).

## Materials and Methods

### Participants and sample collection

The CKD screening program was conducted in a rural population between January 2017 and May 2018 in the Donchang and Kok Samran sub-districts, Khon Kaen Province, Thailand by the Chronic Kidney Disease Northeastern Thailand (CKDNET) program (Cha’on *et al.*, 2020). Seventy-two age- and sex-matched volunteers were recruited. Detailed clinical parameters are shown in Table 1. Participants in the early stages (1–3a) of CKD had a clinically proven impaired kidney structure or renal function as detected by ultrasonography and eGFR values. CKD stage 1 was defined as eGFR ≥90‍ ‍mL‍ ‍min^–1^ 1.73 m^–2^, CKD stage 2 as eGFR of 60–89‍ ‍mL‍ ‍min^–1^ 1.73 m^–2^, and CKD stage 3a as eGFR of 45–59‍ ‍mL‍ ‍min^–1^ 1.73 m^–2^. UD, including diabetes, hypertension, and obesity, are risk factors for CKD. Following the classification from the WHO Regional Office for the Western Pacific (WPRO) (Jitnarin *et al.*, 2011), individuals with BMI >25‍ ‍kg m^–2^ were considered to be obese. Early-stage CKD-UD was defined as the presence of CKD stages 1–3 with a diagnosis of diabetes, hypertension, or other known causes. CKDu was used when no clear cause of the condition was identified: this is a “diagnosis of exclusion” (Cuadra *et al.*, 2006; Floris *et al.*, 2021).

The following exclusion criteria were applied: the current use of antibiotics or probiotics, autoimmune disease, urinary tract infection, and the presence of parasites in stools (confirmed using the modified formalin ethyl-acetate concentration technique and modified agar-plate culture, as previously reported [Kaewrat *et al.*, 2020]). Participants were divided into four groups: healthy controls (HC, *n*=18); those with early-stage CKD of unknown etiology (CKDu, *n*=18); those with early-stage CKD and underlying diseases (CKD-UD, *n*=18), and those free of CKD, but with underlying diseases (UD, *n*=18).

Feces and blood were collected individually from each participant. All fecal samples were kept at –20°C and DNA was extracted within one month. After each participant had fasted for 8 to 12 h, blood was collected into sodium fluoride tubes, kept on ice, and transferred to the Faculty of Medicine. Samples were centrifuged at 3,000×*g* at 4°C for 15‍ ‍min. Serum specimens were transferred into a sterile tube and kept at –20°C for later ana­lyses. The Human Ethical Review Committee of Khon Kaen University (HE602043) approved the study protocol. A signed, informed consent form was obtained from all participants in the CKD project (Cha’on *et al.*, 2020).

### DNA extraction and microbial 16S rRNA sequencing

DNA was extracted from individual fecal samples using a stool DNA kit (QIAGEN) following the manufacturer’s instructions and DNA concentrations were measured. An equal quantity of DNA from each of six individuals was pooled (*i.e.* producing three pooled samples for each treatment group). In each pool, the microbial 16S rRNA gene (v3–v4 regions) was amplified by PCR to detect bacterial genes. The primers used were Pro341-F: 5′-CCTACGGGNGGCWGCAG-3′ and Pro802-R; 5′-TACNVGGGTATCTAATCC-3′ (Itthitaetrakool *et al.*, 2016). The conditions for the PCR reaction were as follows: at 94°C for 5‍ ‍min, 94°C for 40‍ ‍s, 52.8°C for 30‍ ‍s, 72°C for 2‍ ‍min, 35 cycles, followed by an incubation at 72°C for 10‍ ‍min as previously described (Gohl *et al.*, 2016). The PCR product (459 bp) from each pool was subjected to next-generation sequencing to assess the microbial composition. Sequencing libraries were generated using the TruSeq^®^ DNA PCR-Free Sample Preparation Kit (Illumina) according to the manufacturer’s recommendations and index codes were added. Libraries were qualified using a Qubit@ 2.0 Fluorometer (Thermo Scientific) and the Agilent Bioanalyzer 2100 system. The library was sequenced on an Illumina platform using a next-generation sequencer (Illumina^®^ NovaSeq 6000^TM^ system). Based on their unique barcodes, 250-bp paired-end reads were assigned to samples and truncated by the removal of the barcode and primer sequences. Paired-end reads were merged using FLASH version 1.2.7, (Magoč and Salzberg, 2011). Quality filtering on the raw tags was performed under specific filtering conditions to obtain high-quality clean tags according to Qiime version 1.7.0. Tags were compared with the reference database (Gold database) using the UCHIME algorithm to detect chimeric sequences (Edgar *et al.*, 2011), which were subsequently removed (Haas *et al.*, 2011). The sequences passing these steps were termed effective tags.

### Analysis of the microbiome

A microbiome ana­lysis was performed with Uparse software version 7.0.1001 using all of the effective tags. Sequences with ≥97% similarity were assigned to the same operational taxonomic unit (OTU) (Edgar, 2013). A representative sequence for each OTU was screened for further annotation. In each representative sequence, Mothur software and the SSUrRNA database of SILVA (version 132_16s) were used for annotation at each taxonomic rank (Threshold: 0.8~1) (Wang *et al.*, 2007). MUSCLE Version 3.8.31 was used to prepare multiple sequence alignments (Edgar, 2004). The abundance of each OTU was normalized using a standard corresponding to the sample with the fewest sequences before being further analyzed for alpha and beta diversities. ANOSIM and ADONIS ana­lyses were used to test whether two or more groups of samples were significantly different. The distribution of the 35 most abundant genera in samples was displayed in a species abundance heatmap using the R-pheatmap library with Z-score values. Relative abundance data were subjected to Z-score normalization by row. The absolute value of ‘Z’ represents the distance between the raw score and the mean of the standard deviation. The ‘Z’ score is positive when the row abundance is higher than the mean and vice versa. All sequences have been deposited in Genbank (accession numbers OK161038–OK161076). Raw data are available at Mendeley data (doi: https://doi.org/10.17632/7n2cyd2krj.1).

### Measurement of SCFAs using gas chromatography-mass spectrometry (GC-MS)

Serum SCFAs were detected using GC-MS as previously described with slight modifications (Fellows *et al.*, 2018). Briefly, 100‍ ‍μL of each serum sample was added to 1.5-mL microtubes, followed by 20‍ ‍mg of NaCl, 10‍ ‍mg of citric acid, 20‍ ‍μL of 1 M HCl, and 100‍ ‍μL of butanol. The tubes were vortexed for 2‍ ‍min and centrifuged at 18,000×*g* for 15‍ ‍min. The supernatant was transferred to fresh microtubes for later ana­lyses by GC. SCFAs were analyzed using GC-MS (Agilent 7890A GC-7000 Mass Triple Quad) equipped with a capillary column (DB-WAX, 60 m×0.25‍ ‍mm×0.25‍ ‍μm; J&W Scientific) and a quadrupole mass detector. The injector was operated at 260°C in the split mode with a split ratio of 25:1. The volatile free fatty-acid mix standard (acetic acid, butyric acid, formic acid, heptanoic acid, hexanoic acid, isobutyric acid, isovaleric acid, 4-methylvaleric acid, propionic acid, and valeric acid) was purchased from Sigma-Aldrich (Pace *et al.*, 2018, CRM46975). Two microliters of the sample supernatant was injected. Helium was used as the carrier gas with a constant flow rate of 1.0‍ ‍mL‍ ‍min^–1^. The GC oven temperature was started at 40°C for 10‍ ‍min and was increased to 160°C at 3°C min^–1^ and then to 240°C at 10°C min^–1^ (held for 10‍ ‍min). The mass spectrometer was used in the electron ionization mode with the ion-source temperature set at 250°C and ionization energy set at 70 eV. The scan mode was used across a range 30 to 500 m/z. Agilent MassHunter Qualitative Analysis B.04.00 software was used for data ana­lyses. The identification of SCFAs was performed by comparing mass spectra with NIST mass-spectral libraries (National Institute of Standards, 2011 version).

### Statistical ana­lysis

Non-parametric statistical ana­lyses or Pearson’s chi-square test were used to investigate the significance of differences in index variables between groups. A one-way ana­lysis of variance was performed to compare clinical parameters between groups. Welch’s *t*-test was used to identify species showing a significantly different relative abundance between groups at various taxonomic ranks (phylum, genus, and species). A *t*-test was also used to compare the average age of patients between groups. The relative abundance of the top 35 genera was compared between pairs of groups using a multiple *t*-test ana­lysis. The results obtained were displayed in a volcano plot. GraphPad Prism 8 (GraphPad Software) was used for statistical ana­lyses. A *P*-value or adjusted *P*-value <0.05 was considered to be significant. The linear discriminant ana­lysis effect size (LEfSe) was used to identify the taxonomic units most likely to explain differences between classes by coupling standard tests for the significance of differences with additional tests encoding biological consistency and effect relevance (Segata *et al.*, 2011).

## Results

### Diversity of the gut microbiome in study groups

**Fig. 1.**
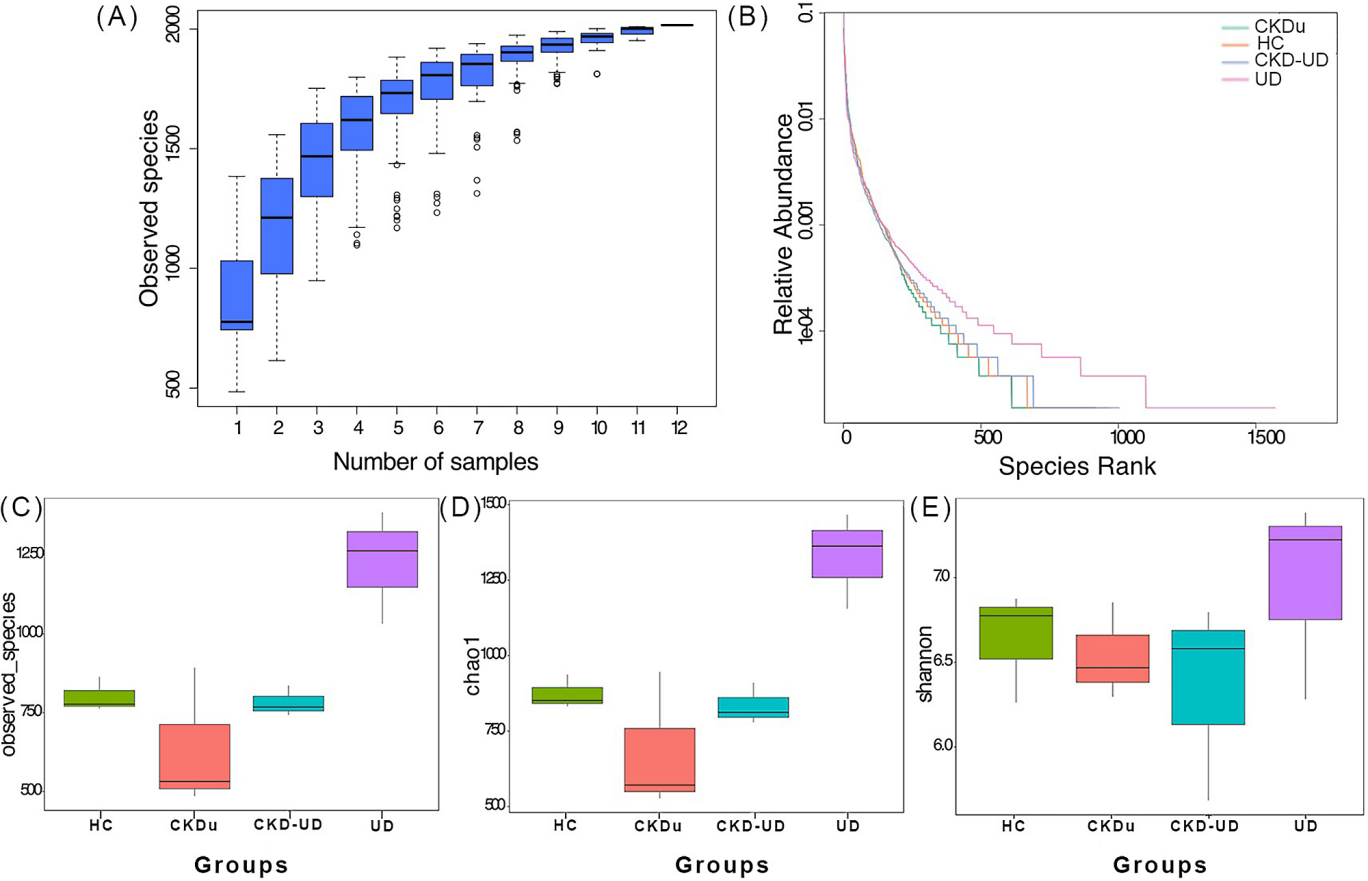
Gut microbial alpha diversity in patient groups (CKD, CKD-UD, and UD) and the healthy control group. (A) Species accumulation curves, (B) rank abundance curves, (C) numbers of observed species, (D) the Chao1 richness index, and (E) the Shannon index.

Following filtering, an average of 71,653 high-quality sequences was obtained for each sample. The species accumulation curve showed that sufficient sequencing had been performed to identify most of the species present (Fig. 1A). We investigated species richness and evenness using a rank-abundance curve. The steep gradient for each sample indicated that some taxa were present at a high abundance (Fig. 1B). The observed numbers of OTUs in each patient group are shown in Fig. 1C. The Chao1 index reflects the total richness of microbial communities (Fig. 1D), while the Shannon index indicates the average abundance of different species in a sample (Fig. 1E). The results obtained showed that the UD group had the highest alpha diversity followed by the HC group and then the CKD-UD and CKDu groups.

**Fig. 2.**
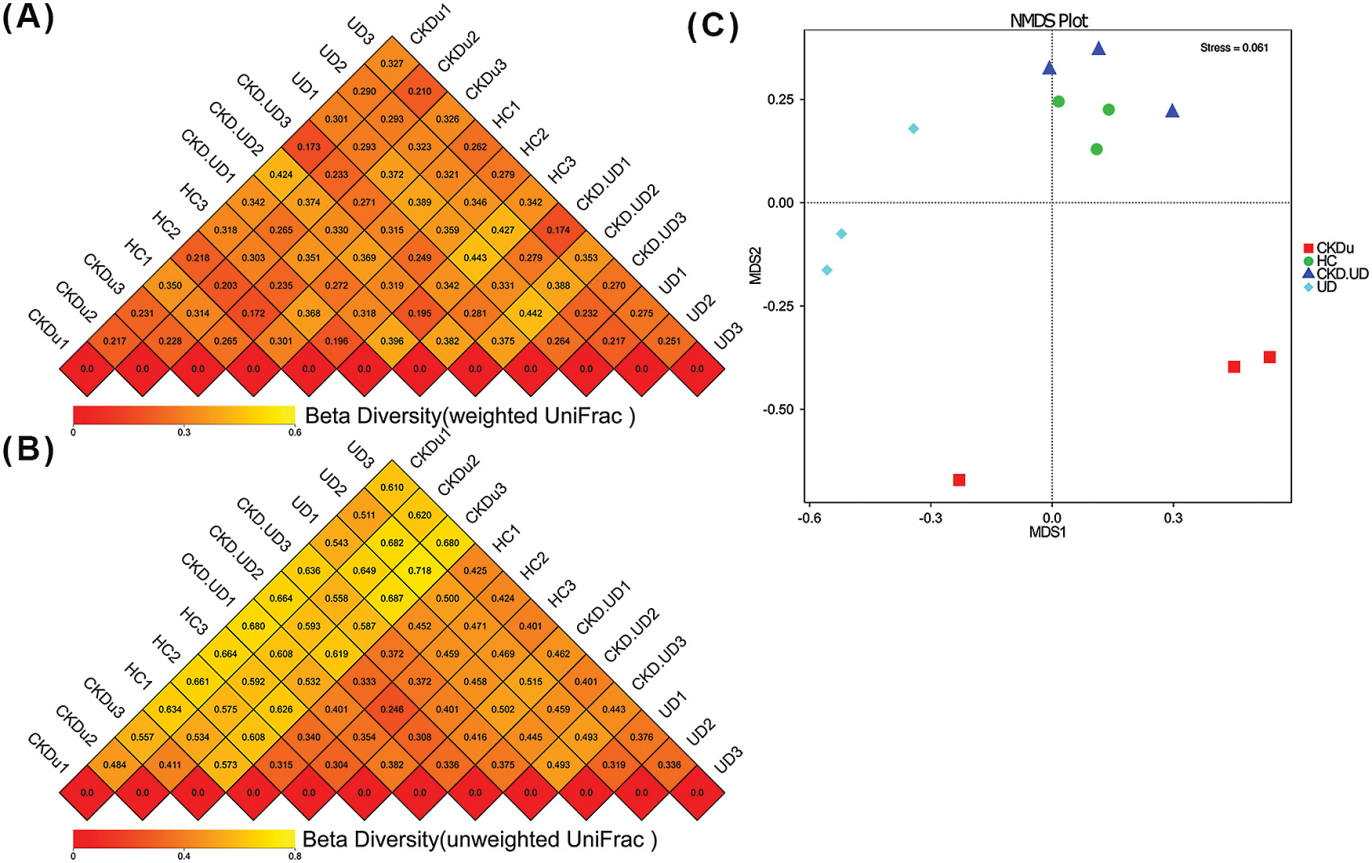
Beta diversity ana­lysis of patient groups and the healthy control group. (A) A heatmap of weighted UniFrac and (B) unweighted UniFrac distances. The numbers in cells represent the pairwise difference in coefficient values between samples. Colors also indicate the degree of the difference (color scale at the bottom). (C) A non-metric multidimensional scaling (NMDS) plot in which green represents the healthy control group, red represents the CKD group, navy represents the CKD-UD group, and light blue represents the UD group.

Beta diversity is displayed using a heatmap based on weighted UniFrac (Fig. 2A) and unweighted UniFrac distances (Fig. 2B) to compare the dissimilarity coefficient among all pairs of samples. Non-metric multidimensional scaling (NMDS) provided the diversity patterns of microbial communities from different sample groups (Fig. 2C). The results obtained revealed that the diversity profile of the HC group was clearly distinct from those of the CKDu and UD groups, but not from that of the CKD-UD group. The ANOSIM statistic, comparing the mean of ranked dissimilarities between groups to the mean of ranked dissimilarities within groups, was applied, but did not detect any dissimilarity. ADONIS testing showed a significant difference only between the CKDu and CKD-UD groups (R^2^=0.26158, Pr[>F]=0.0014).

### Gut dysbiosis in early stages of CKD

**Fig. 3.**
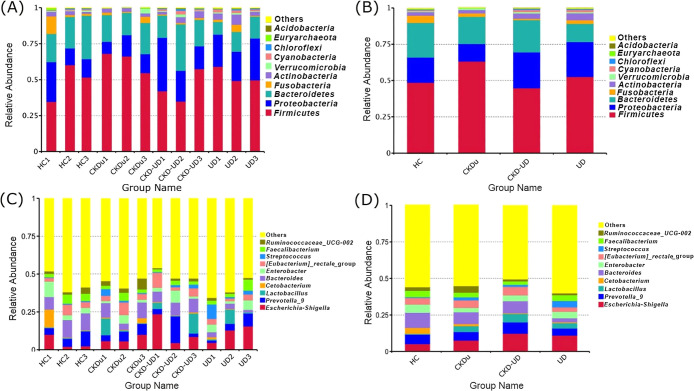
Relative abundance of bacterial taxa in patient groups and the healthy control group. (A) Each individual pool (three per group) and (B) groups of subjects at the phylum level. (C) Each individual pool and (D) groups of subjects at the genus level. “Others” represents all taxa besides the top 10.

Sequence reads sharing >97% similarity were grouped into the same OTU. The numbers of sequences representing each OTU indicated the relative abundance of each taxon in‍ ‍each patient group and the HC group. *Firmicutes*, *Bacteroidetes* and *Proteobacteria*, in descending order, were the most abundant phyla and also within the CKDu group (Fig. 3A and B).

At the genus level, there was an increased abundance (relative to healthy controls) of *Escherichia-Shigella* and *Lactobacillus* and decreased abundance of *Bacteroides*, *Enterobacter*, *Cetobacterium*, and *Faecalibacterium* in the CKDu, UD, and CKD-UD groups. In comparisons with the CKDu group, the abundance of *Escherichia-Shigella* and *Enterobacter* was higher in the CKD-UD and UD groups, while that of *Bacteroides*, *Ruminococcaceae_UCG-002*, and *[Eubacterium]_rectale_*group was lower in the UD group (Fig. 3C and D).

**Fig. 4.**
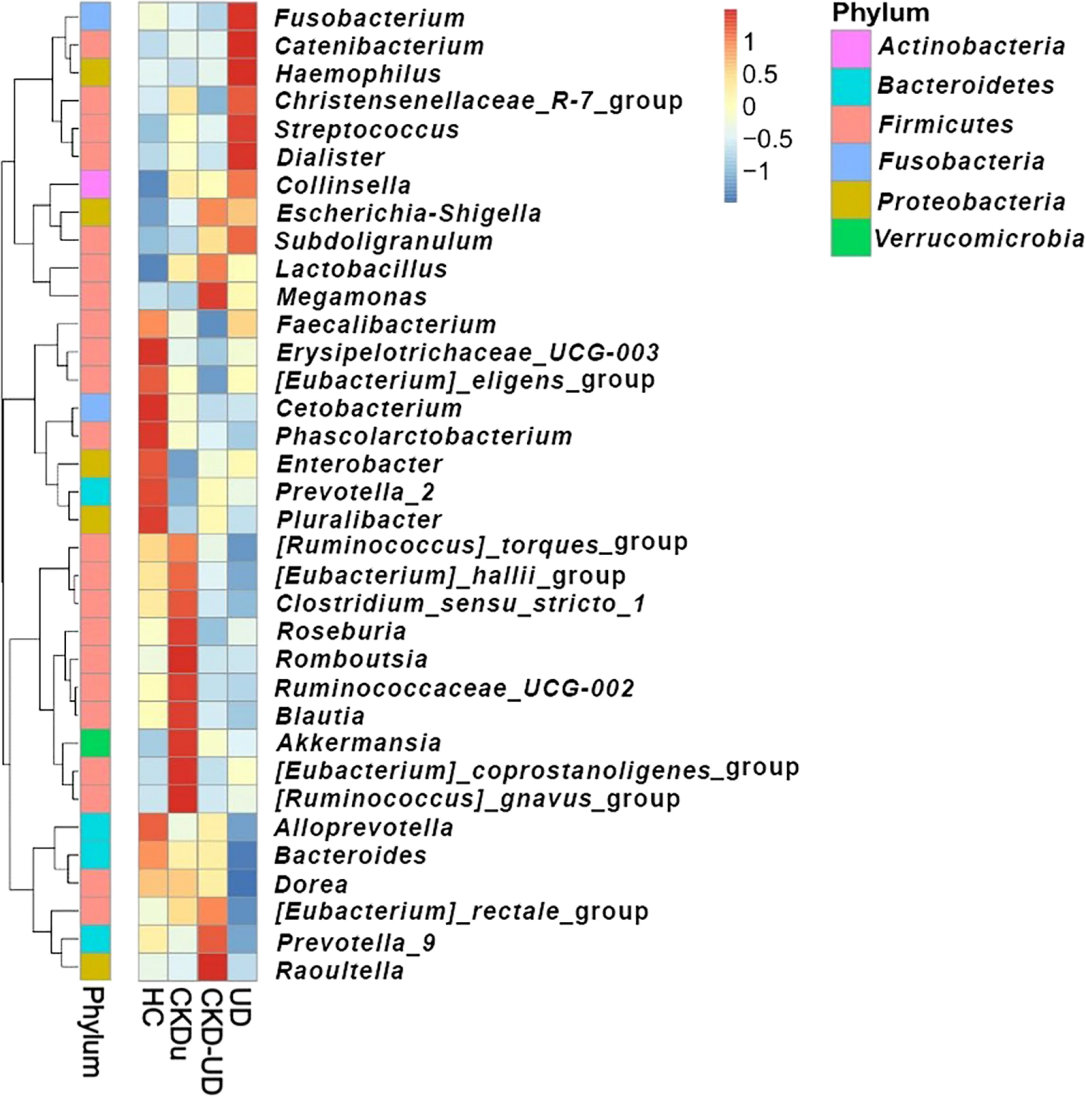
Taxonomic heatmap showing the relative abundance of 35 bacterial genera in various groups. The different colors represent different z-score values. Shades of blue indicate low abundance (z-score: –1 to 0) and shades of red indicate high abundance, z-score: 0 to 1.

**Fig. 5.**
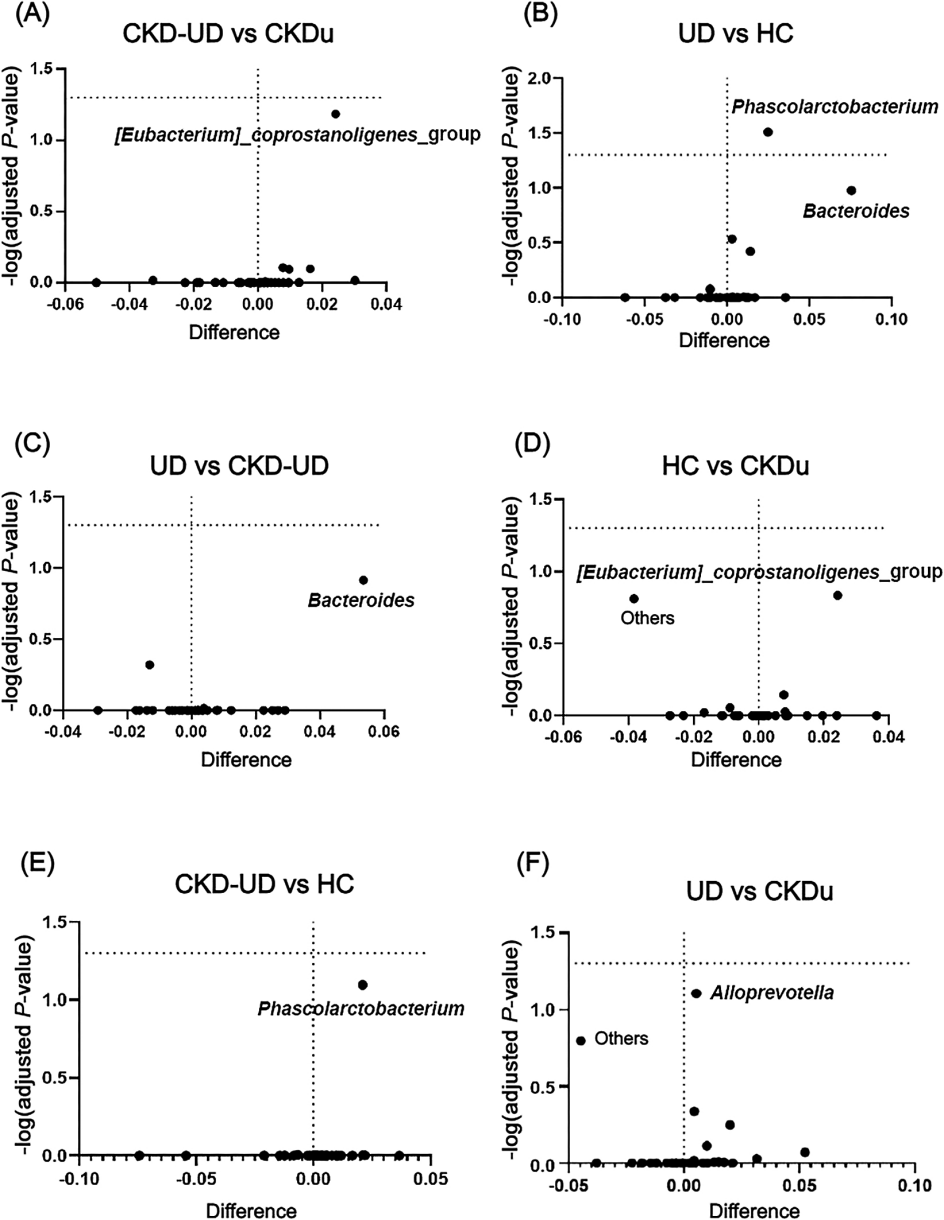
Volcano plots identifying genera (dots, some labeled) that significantly differ in abundance between pairs of groups. The dots labeled with taxon names are discussed in the text. (A) *[Eubacterium]_coprostanoligenes_*group between CKD-UD vs CKDu, (B) *Phascolarctobacterium* and *Bacteroides* between UD vs HC, (C) *Bacteroides* UD vs CKD-UD, (D) *[Eubacterium]_coprostanoligenes_*group between HC vs CKDu, (E) *Phascolarctobacterium* CKD-UD vs HC, and (F) *Alloprevotella* between UD vs CKDu.

**Fig. 6.**
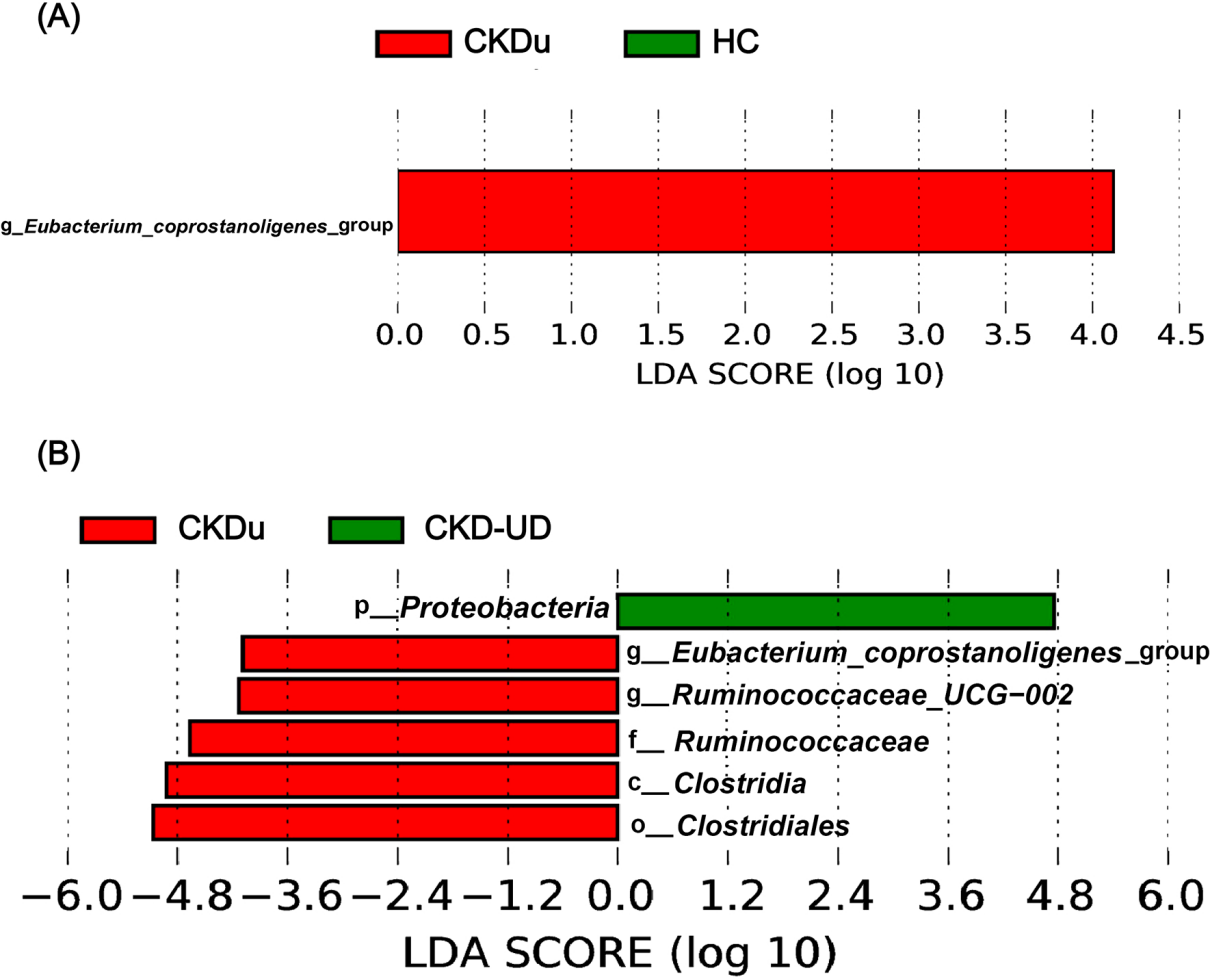
Linear discriminant ana­lysis (LDA) effect size (LEfSe) was used to identify taxa that best discriminated between pairs of groups. LDA score >2.0 at taxonomic levels from phylum to species. (A) CKDu versus HC, (B) CKDu versus CKD-UD. Red bars indicate that a taxon is more abundant in the CKDu group and green bars that it is more abundant in the CKD-UD group.

The heatmap in Fig. 4 shows the relative abundance of the 35 most common genera. *Faecalibacterium*, *Erysipelotrichaceae_UCG–003*, *[Eubacterium]_eligens_group*, *Cetobacterium*, *Phascolarctobacterium*, *Enterobacter*, *Prevotella_2*, *Pluralibacter*, *Alloprevotella*, *Bacteroides*, and *Dorea* were abundant in the HC group. *[Ruminococcus]_torques_*group, *[Eubacterium]_hallii_*group, *Clostridium_sensu_stricto_1*, *Roseburia*, *Romboutsia*, *Ruminococcaceae_UCG–002*, *Blautia*, *Akkermansia*, *[Eubacterium]_coprostanoligenes_*group, and *[Ruminococcus]_gnavus_*group were abundant in the CKDu group. *Escherichia–Shigella*, *Subdoligranulum*, *Lactobacillus*, *Megamonas*, *[Eubacterium]_rectale_*group, *Prevotella_9*, and *Raoultella* were abundant in the CKD-UD group.‍ ‍*Fusobacterium*, *Catenibacterium*, *Haemophilus*, *Christensenellaceae_R–7_group*, *Streptococcus*, *Dialister*, *Collinsella*, *Escherichia–Shigella*, and *Subdoligranulum* were abundant in the UD group. The results of statistical ana­lyses indicated that different genera were included in the top 35 in comparisons between different pairs of groups (Fig. 5). In comparisons with the HC group, the abundance of *Phascolarctobacterium* was significantly lower in groups with underlying diseases (UD vs HC, adjusted *P*-value=0.031; CKD-UD vs HC, adjusted *P*-value=0.08). The abundance of *Bacteroides* was slightly lower in the UD group than in the HC and CKD-UD groups (UD vs HC, adjusted *P*-value=0.105; UD vs CKD-UD, adjusted *P*-value=0.122). *[Eubacterium]_coprostanoligenes_*group was more abundant in the CKDu group than in the HC group (adjusted *P*-value=0.147) and CKD-UD group (adjusted *P*-value=0.065). In addition, LEfSe identified which taxa were most likely to explain differences between the CKDu and HC groups (Fig. 6A) and between the CKDu and CKD-UD groups (Fig. 6B). In both of these comparisons, the abundance of *[Eubacterium]_coprostanoligenes_*group was significantly higher in the CKDu group than in the HC and CKD-UD groups.

### Reduction in serum concentrations of SCFAs in early stages of CKD

**Fig. 7.**
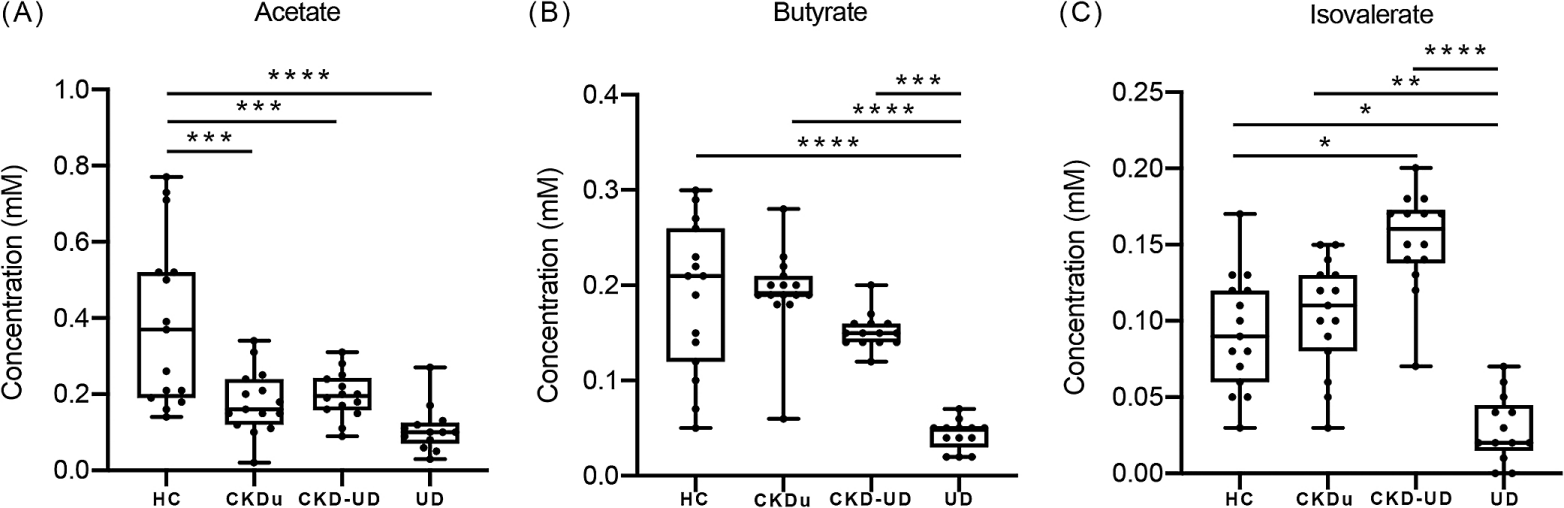
Serum concentrations of short-chain fatty acids in each group. (A) Acetate, (B) butyrate, and (C) isovalerate. The dots represent individual subjects in the HC group (*n*=15), CKD group (*n*=15), CKD-UD group (*n*=14), and UD group (*n*=13). The concentration of each SCFA was compared between groups using a one-way ana­lysis of variance, *P<0.05, ** *P*<0.01, *** *P*<0.001, **** *P*<0.0001.

We compared serum concentrations of SCFAs in the healthy group (HC) and the patient (CKDu, CKD-UD, and‍ ‍UD) groups. The SCFAs identified in serum samples were acetate, butyrate, and isovalerate (Fig. 7). Acetate concentrations were significantly lower in the CKDu (0.1793‍ ‍mM‍ ‍L^–1^), CKD-UD (0.1964‍ ‍mM L^–1^), and UD groups (0.1085‍ ‍mM L^–1^) than in the healthy control group (0.3907‍ ‍mM L^–1^), (*P*=0.001, *P*=0.001, and *P*=0.0001, respectively, Fig. 7A). Butyrate and isovalerate concentrations were significantly lower in the UD group than in the other groups (Fig. 7B and C). However, acetate, butyrate, and isovalerate concentrations were similar between the CKDu and CKD-UD groups (Fig. 7).

## Discussion

We herein investigated, for the first time, the gut microbiota profile in early-stage CKDu in the absence or presence of other metabolic diseases that have been identified as risk factors for CKD in order to elucidate the relationship between gut dysbiosis and CKD in two communities in Khon Kaen Province, Northeast Thailand. Although we did not control for differences in diet, all participants resided in the same sub-district and matched pairs of participants were used. We employed three sets of pooled samples in each group to allow for statistical ana­lyses. We identified minor changes in the gut microbiome and significantly lower serum concentrations of acetate in the CKDu, CKD-UD, and UD groups than in the HC group, suggesting that decreased acetate concentrations are associated with a wide range of disease states, not just CKD. Among the changes observed in the gut microbiome, *[Eubacterium]_coprostanoligenes*_group was more abundant in the CKDu group than in the HC and CKD-UD groups. Furthermore, the lowest serum concentrations of acetate, butyrate and isovalerate were detected in the groups with underlying diseases, such as diabetes type 2 and hypertension. Awareness that SCFA concentrations are reduced in CKD and a wide range of other diseases provides support for the use of these compounds as dietary supplements to limit disease progression (Feng *et al.*, 2018; Alsharairi, 2022; Caetano and Castelucci, 2022; Yao *et al.*, 2022).

Gut dysbiosis was noted in the CKDu, CKD, and UD groups. Alpha diversity was slightly decreased in the CKD-UD and CKDu groups, but was the highest in the UD group. The CKDu and UD groups had gut microbiome communities that were distinct from that of the HC group: however, the ADONIS ana­lysis showed that the microbiome community in the CKDu group significantly differed from that in the CKD-UD group. The result showing gut microbial diversity in early-stage CKDu was consistent with previous findings on late-stage CKD patients with or without hemodialysis (Li *et al.*, 2019). The present study revealed that the most abundant bacterial phyla in each study group were *Firmicutes*, *Bacteroidetes*, and *Proteobacteria*. This is in accordance with previous findings on the gut microbiomes of healthy controls and CKD patients (Lun *et al.*, 2019). At the phylum level, *Firmicutes* were more abundant, while *Bacteroidetes* were less abundant in the CKDu group than in the HC group. The reduction observed in the abundance of *Bacteroidetes* was consistent with previous findings on advanced-stage CKD in China (Huang *et al.*, 2022), but was in contrast to those on CKD in dialysis patients in Taiwan (Shivani *et al.*, 2022). This discrepancy may be attributed to the different geographies and characteristics of subjects examined (Arumugam *et al.*, 2011).

The early detection of CKD is challenging due to the absence of early-stage symptoms. Therefore, we attempted to identify the features of the gut microbiome that may be affected in CKD stages 1–3 and in the presence of underlying diseases, including diabetes mellitus, obesity, and hypertension, all known risk factors for the progression of CKD. In the present study, the gut microbiome of the UD group was distinct from those of the HC, CKDu, and CKD-UD groups. Bacterial taxa enriched in the UD group including the genera *Fusobacterium*, *Escherichia-shigella*, and *Subdoligranulum*. Previous studies demonstrated that these taxa were more abundant in kidney disease patients (Jiang *et al.*, 2017). Moreover, several taxa of gut microbes have been linked to numerous clinical markers in kidney patients. *Fusobacterium* and *Escherichia-Shigella* positively correlate with increased renal inflammation markers, including cystatin C and serum creatinine in ESRD patients (Jiang *et al.*, 2017). Similarly, a positive correlation has been reported between the abundance of *Escherichia* and *Fusobacterium* and serum uremic toxin trimethylamine *N*-oxide levels as well as indoxyl sulphate and p-cresyl sulfate levels, which have been associated with reduced kidney function (measured by eGFR) (Al-Obaide *et al.*, 2017; Wang *et al.*, 2020). However, the present study did not detect a correlation between gut microbial profiles and renal function markers due to the use of pooled samples; therefore, an ana­lysis of individual samples is warranted in the future.

Intestinal tight-junction proteins are depleted during the pathogenesis of kidney diseases, leading to changes in gut permeability in association with kidney fibrosis (Sabatino *et al.*, 2015; Chen *et al.*, 2019). Probiotic bacteria may contribute to the preservation of intestinal barrier functions through the production of SCFAs, which are an important nutrient source for enterocytes and also moderate immune responses (Yao *et al.*, 2022). The present results revealed that the abundance of SCFA-producing bacteria, including *Bacteroides*, was significantly reduced in the UD group. These results suggest that a reduction in *Bacteroides* associated with underlying diseases is a risk factor for the development of CKD, which was supported by findings obtained on CKD patients (Huang *et al.*, 2022) and CKD (stages 3–5) patients receiving colonic dialysis (Li *et al.*, 2021). In addition, the abundance of *Phascolarctobacterium* was significantly reduced in the UD and CKD-UD groups, which is consistent with previous findings on patients with end-stage CKD (Hu *et al.*, 2022).

The term CKDu refers to a type of CKD that primarily affects marginalized agricultural communities in particular parts of the world where a large number of individuals develop an undiagnosed, fatal form of kidney disease (Weaver *et al.*, 2015). CKDu has been attracting increasing attention in the scientific community because the affected populations are some of the poorest in the world and are exposed to many occupational and environmental dangers. In the present study, the abundance of *[Eubacterium] coprostanoligenes*_group was higher in the CKDu group than in the HC and CKD-UD groups. This bacterial taxon is a cholesterol-reducing microorganism (Freier *et al.*, 1994). Other studies identified this genus in Alzheimer’s dementia patients (Kaiyrlykyzy *et al.*, 2022) and demonstrated that it significantly protected against colitis and carcinogenesis in the colons of mice (Bai *et al.*, 2021). However, we herein showed for the first time an increase in the abundance of this taxon in human CKD. Therefore, an increased abundance of this genus in individuals without an apparent underlying disease and in regions of poor water quality and chemical exposure indicates that they need to be checked for early-stage CKDu. Further studies are needed on the relationship between environmental pollutants (such as heavy metals) and the gut microbiota of CKDu patients.

We investigated serum SCFAs and identified acetate, butyrate, and isovalerate. SCFAs are produced by the bacterial fermentation of dietary fiber and resistant starch in the intestines and play many roles in human physiology, such as anti-inflammation, energy regulation, and the maintenance of intestinal barrier functions (Zeng *et al.*, 2019; Magliocca *et al.*, 2022; Yao *et al.*, 2022). In the present study, acetate concentrations were significantly lower in the early-stage CKDu group than in the healthy control group. Reductions in the concentrations of SCFAs, including acetate, butyrate, and propionate, were previously demonstrated in CKD patients (Wang *et al.*, 2019; Lu *et al.*, 2021). The UD group exhibited the most significant reduction in SCFA-producing bacteria and, thus, acetate, butyrate, and isovalerate concentrations. The depletion of SCFAs observed in the early stages of CKDu may permit the translocation of bacterial toxins to the kidney (Lau *et al.*, 2018). Decreases in serum concentrations of acetate, but not butyrate in CKDu may be explained by the enriched abundance of butyrate due to the presence of *[Eubacterium]* spp. (Mukherjee *et al.*, 2020). Serum acetate concentrations in healthy individuals in a rural area of Khon Kaen were similar to those in Swedish subjects (Olsson *et al.*, 2021), but were higher than those in urban Canadians (Wolever *et al.*, 1996), suggesting that differences in diet, individual status, and measurement methods affected the findings obtained (Moffett *et al.*, 2020).

It is postulated that CKDu is multifactorial, involving genetic predisposition, nutritional stress, and the hydration status (Vlahos *et al.*, 2019). In our previous study on two communities, the majority of participants worked on farms growing crops, such as sugar cane, which is associated with high pesticide use (Cha’on *et al.*, 2020). Further studies are required to obtain a more detailed understanding of these and other possible risk factors and their effects on the gut microbiome. Studies on environmental pollution (Xu *et al.*, 2018) and chemical contaminants in water (McDonough *et al.*, 2020) are needed to detect any relationship with the gut microbiome in CKDu. The use of pooled DNA samples is a pragmatic solution to the cost of sequencing (Ray *et al.*, 2019), but means that information on individual variations may be obscured. This prevented us from performing a correlation ana­lysis between the clinical parameters and SCFAs of patients.

## Conclusion

The present results revealed that early-stage CKDu was associated with an increased relative abundance of bacteria and decreased concentrations of SCFAs. Most of the changes observed in the gut microbiome in CKDu were consistent with previous findings on late-stage CKD. Reductions in the serum concentrations of acetate and the increased abundance of *[Eubacterium]_coprostanoligenes_*group in early-stage CKDu may be useful for screening at-risk individuals. This may also facilitate measures to prevent the progression of CKD to ESRD in these individuals. The identification of environmental toxicants associated with the development of CKDu in this region of Thailand is now a priority for future work.

## Citation

Banjong, D., Pongking, T., Tran, N. T.. D.., Pinlaor, S., Dangtakot, R., Intuyod, K., et al. (2023) Slight Changes in the Gut Microbiome in Early-stage Chronic Kidney Disease of Unknown Etiology. *Microbes Environ ***38**: ME22097.

https://doi.org/10.1264/jsme2.ME22097

## Figures and Tables

**Table 1.** Clinical parameters (mean and [SD]) and comparisons of patient groups and healthy controls.

Biochemical tests and other factors	HC (*n*=18)	CKDu (*n*=18)	CKD-UD (*n*=18)	UD (*n*=18)
Sex (Male: Female)	9:9	9:9	5:13	5:13
Age (years)	62.72 (11.83)	63.22 (14.95)	64.22 (9.78)	63.89 (7.66)
Body mass index (kg m^–2^)	21.86 (2.06)	20.33 (2.21)	28.37 (3.27)****	29.78 (4.06)****
Serum creatinine (mg dL^–1^)	0.82 (0.18)	0.98 (0.20)	0.97 (0.30)	0.77 (0.13)
Urine creatinine (mg dL^–1^)	107.90 (52.31)	115.90 (56.60)	116.20 (76.38)	103.40 (50.00)
eGFR (mL‍ ‍min^–1^ 1.73 m^–2^)	95.61 (10.26)	73.98 (19.45)**	71.69 (20.53)***	83.68 (14.48)
Microalbumin (mg dL^–1^)	1.03 (0.81)	2.36 (4.43)	9.11 (16.08)*	4.438 (9.86)
Uric Acid (mg dL^–1^)	5.17 (1.15)	5.45 (1.70)	5.94 (1.51)	5.32 (1.04)
ALT (IU L^–1^)	26.05 (15.43)	19.74 (8.51)	26.87 (22.84)	34.31 (25.39)
Glucose (mg dL^–1^)	87.72 (8.42)	89.22 (11.73)	167.60 (53.58)***	138.20 (44.98)***
HbA1C	5.52 (0.33)	5.56 (0.35)	8.54 (2.04)****	8.11 (1.43)****
LDL Cholesterol (mg dL^–1^)	115.90 (30.48)	121.40 (39.19)	122.50 (39.73)	109.80 (27.18)
Systolic blood pressure (mmHg)	117.4 (11.13)	120.7 (12.12)	152.4 (16.02)****	140.0 (14.56)****
Diastolic blood pressure (mmHg)	73.17 (9.685)	76.06 (9.735)	87.06 (9.428)***	83.28 (9.08)**

Abbreviations: Healthy control (HC), early-stage CKD (CKD), early-stage CKD combined with underlying diseases of CKD (CKD-UD), and underlying diseases of CKD (UD). Significantly different from the healthy group using a one-way ana­lysis of variance, * *P*<0.05, ** *P*<0.01, *** *P*<0.001, **** *P*<0.0001.

## References

[B1] Aekplakorn, W., Chariyalertsak, S., Kessomboon, P., Assanangkornchai, S., Taneepanichskul, S., Neelapaichit, N., et al. (2021) Women and other risk factors for chronic kidney disease of unknown etiology in Thailand: National Health Examination V Survey. Sci Rep 11: 21366. 34725395 10.1038/s41598-021-00694-9 PMC8560950

[B2] Al-Obaide, M.A.I., Singh, R., Datta, P., Rewers-Felkins, K.A., Salguero, M.V., Al-Obaidi, I., et al. (2017) Gut microbiota-dependent trimethylamine-N-oxide and serum biomarkers in patients with T2DM and advanced CKD. J Clin Med 6: 86. 28925931 10.3390/jcm6090086 PMC5615279

[B3] Alsharairi, N.A. (2022) The therapeutic role of short-chain fatty acids mediated very low-calorie ketogenic diet-gut microbiota relationships in paediatric inflammatory bowel diseases. Nutrients 14: 4113. 36235765 10.3390/nu14194113 PMC9572225

[B4] Arumugam, M., Raes, J., Pelletier, E., Le Paslier, D., Yamada, T., Mende, D.R., et al. (2011) Enterotypes of the human gut microbiome. Nature 473: 174–180. 21508958 10.1038/nature09944 PMC3728647

[B5] Bai, D., Sun, T., Zhao, J., Du, J., Bu, X., Cao, W., et al. (2021) Oroxylin A maintains the colonic mucus barrier to reduce disease susceptibility by reconstituting a dietary fiber-deprived gut microbiota. Cancer Lett 515: 73–85. 34052330 10.1016/j.canlet.2021.05.018

[B6] Boonjaraspinyo, S., Boonmars, T., Kaewsamut, B., Ekobol, N., Laummaunwai, P., Aukkanimart, R., et al. (2013) A cross-sectional study on intestinal parasitic infections in rural communities, northeast Thailand. Korean J Parasitol 51: 727–734. 24516280 10.3347/kjp.2013.51.6.727 PMC3916464

[B7] Caetano, M.A.F., and Castelucci, P. (2022) Role of short chain fatty acids in gut health and possible therapeutic approaches in inflammatory bowel diseases. World J Clin Cases 10: 9985–10003. 36246826 10.12998/wjcc.v10.i28.9985 PMC9561599

[B8] Cha’on, U., Wongtrangan, K., Thinkhamrop, B., Tatiyanupanwong, S., Limwattananon, C., Pongskul, C., et al. (2020) CKDNET, a quality improvement project for prevention and reduction of chronic kidney disease in the Northeast Thailand. BMC Public Health 20: 1299. 32854662 10.1186/s12889-020-09387-w PMC7450931

[B9] Chen, Y.Y., Chen, D.Q., Chen, L., Liu, J.R., Vaziri, N.D., Guo, Y., and Zhao, Y.Y. (2019) Microbiome-metabolome reveals the contribution of gut-kidney axis on kidney disease. J Transl Med 17: 5. 30602367 10.1186/s12967-018-1756-4 PMC6317198

[B10] Cuadra, S., Jakobsson, K., Hogstedt, C., and Wesseling, C. (2006) Chronic kidney disease: Assessment of current knowledge and feasibility for regional research collaboration in Central America. Heredia, Costa Rica: Program on Work and Health in Central America (SALTRA), Central American Institute for Studies on Toxic Substances (IRET-UNA).

[B11] Edgar, R.C. (2004) MUSCLE: multiple sequence alignment with high accuracy and high throughput. Nucleic Acids Res 32: 1792–1797. 15034147 10.1093/nar/gkh340 PMC390337

[B12] Edgar, R.C., Haas, B.J., Clemente, J.C., Quince, C., and Knight, R. (2011) UCHIME improves sensitivity and speed of chimera detection. Bioinformatics 27: 2194–2200. 21700674 10.1093/bioinformatics/btr381 PMC3150044

[B13] Edgar, R.C. (2013) UPARSE: highly accurate OTU sequences from microbial amplicon reads. Nat Methods 10: 996–998. 23955772 10.1038/nmeth.2604

[B14] Fellows, R., Denizot, J., Stellato, C., Cuomo, A., Jain, P., Stoyanova, E., et al. (2018) Microbiota derived short chain fatty acids promote histone crotonylation in the colon through histone deacetylases. Nat Commun 9: 105. 29317660 10.1038/s41467-017-02651-5 PMC5760624

[B15] Feng, W., Ao, H., and Peng, C. (2018) Gut microbiota, short-chain fatty acids, and herbal medicines. Front Pharmacol 9: 1354. 30532706 10.3389/fphar.2018.01354 PMC6265305

[B16] Floris, M., Lepori, N., Angioi, A., Cabiddu, G., Piras, D., Loi, V., et al. (2021) Chronic kidney disease of undetermined etiology around the world. Kidney Blood Pressure Res 46: 142–151. 10.1159/000513014 33845480

[B17] Freier, T.A., Beitz, D.C., Li, L., and Hartman, P.A. (1994) Characterization of *Eubacterium coprostanoligenes* sp. nov., a cholesterol-reducing anaerobe. Int J Syst Bacteriol 44: 137–142. 8123557 10.1099/00207713-44-1-137

[B18] GBD 2013 Mortality and Causes of Death Collaborators. (2015) Global, regional, and national age-sex specific all-cause and cause-specific mortality for 240 causes of death, 1990–2013: a systematic ana­lysis for the Global Burden of Disease Study 2013. Lancet 385: 117–171. 25530442 10.1016/S0140-6736(14)61682-2 PMC4340604

[B19] Gohl, D.M., Vangay, P., Garbe, J., MacLean, A., Hauge, A., Becker, A., et al. (2016) Systematic improvement of amplicon marker gene methods for increased accuracy in microbiome studies. Nat Biotechnol 34: 942–949. 27454739 10.1038/nbt.3601

[B20] Haas, B.J., Gevers, D., Earl, A.M., Feldgarden, M., Ward, D.V., Giannoukos, G., et al. (2011) Chimeric 16S rRNA sequence formation and detection in Sanger and 454-pyrosequenced PCR amplicons. Genome Res 21: 494–504. 21212162 10.1101/gr.112730.110 PMC3044863

[B21] Hai, N.T., Hongsrichan, N., Intuyod, K., Pinlaor, P., Yingklang, M., Chaidee, A., et al. (2022) *Strongyloides stercoralis* infection induces gut dysbiosis in chronic kidney disease patients. PLoS Neglected Trop Dis 16: e0010302. 10.1371/journal.pntd.0010302 PMC9481163 36067216

[B22] Haonon, O., Liu, Z., Dangtakot, R., Intuyod, K., Pinlaor, P., Puapairoj, A., et al. (2021) *Opisthorchis viverrini* infection induces metabolic and fecal microbial disturbances in association with liver and kidney pathologies in hamsters. J Proteome Res 20: 3940–3951. 34270897 10.1021/acs.jproteome.1c00246

[B23] Hills, R.D., Jr., Pontefract, B.A., Mishcon, H.R., Black, C.A., Sutton, S.C., and Theberge, C.R. (2019) Gut microbiome: Profound implications for diet and disease. Nutrients 11: 1613. 31315227 10.3390/nu11071613 PMC6682904

[B24] Hobby, G.P., Karaduta, O., Dusio, G.F., Singh, M., Zybailov, B.L., and Arthur, J.M. (2019) Chronic kidney disease and the gut microbiome. Am J Physiol Renal Physiol 316: F1211–F1217. 30864840 10.1152/ajprenal.00298.2018 PMC6620595

[B25] Hu, J., Zhong, X., Liu, Y., Yan, J., Zhou, D., Qin, D., et al. (2022) Correlation between intestinal flora disruption and protein-energy wasting in patients with end-stage renal disease. BMC Nephrol 23: 130. 35369865 10.1186/s12882-022-02762-2 PMC8978364

[B26] Huang, Y., Xin, W., Xiong, J., Yao, M., Zhang, B., and Zhao, J. (2022) The intestinal microbiota and metabolites in the gut-kidney-heart axis of chronic kidney disease. Front Pharmacol 13: 837500. 35370631 10.3389/fphar.2022.837500 PMC8971625

[B27] Ingsathit, A., Thakkinstian, A., Chaiprasert, A., Sangthawan, P., Gojaseni, P., Kiattisunthorn, K., et al. (2010) Prevalence and risk factors of chronic kidney disease in the Thai adult population: Thai SEEK study. Nephrol Dial Transplant 25: 1567–1575. 20037182 10.1093/ndt/gfp669

[B28] Itthitaetrakool, U., Pinlaor, P., Pinlaor, S., Chomvarin, C., Dangtakot, R., Chaidee, A., et al. (2016) Chronic *Opisthorchis viverrini* infection changes the liver microbiome and promotes *Helicobacter* growth. PLoS One 11: e0165798. 27806126 10.1371/journal.pone.0165798 PMC5091914

[B29] Jadoon, A., Mathew, A.V., Byun, J., Gadegbeku, C.A., Gipson, D.S., Afshinnia, F., and Pennathur, S. (2018) Gut microbial product predicts cardiovascular risk in chronic kidney disease patients. Am J Nephrol 48: 269–277. 30326477 10.1159/000493862 PMC6280192

[B30] Jandhyala, S.M., Talukdar, R., Subramanyam, C., Vuyyuru, H., Sasikala, M., and Nageshwar Reddy, D. (2015) Role of the normal gut microbiota. World J Gastroenterol 21: 8787–8803. 26269668 10.3748/wjg.v21.i29.8787 PMC4528021

[B31] Jiang, S., Xie, S., Lv, D., Wang, P., He, H., Zhang, T., et al. (2017) Alteration of the gut microbiota in Chinese population with chronic kidney disease. Sci Rep 7: 2870. 28588309 10.1038/s41598-017-02989-2 PMC5460291

[B32] Jitnarin, N., Kosulwat, V., Rojroongwasinkul, N., Boonpraderm, A., Haddock, C.K., and Poston, W.S. (2011) Prevalence of overweight and obesity in Thai population: results of the National Thai Food Consumption Survey. Eat Weight Disord 16: e242–249. 22526130 10.1007/BF03327467 PMC5824639

[B33] Kaewrat, W., Sengthong, C., Yingklang, M., Intuyod, K., Haonon, O., Onsurathum, S., et al. (2020) Improved agar plate culture conditions for diagnosis of *Strongyloides stercoralis*. Acta Tropica 203: 105291. 31816322 10.1016/j.actatropica.2019.105291

[B34] Kaiyrlykyzy, A., Kozhakhmetov, S., Babenko, D., Zholdasbekova, G., Alzhanova, D., Olzhayev, F., et al. (2022) Study of gut microbiota alterations in Alzheimer’s dementia patients from Kazakhstan. Sci Rep 12: 15115. 36068280 10.1038/s41598-022-19393-0 PMC9448737

[B35] Kazancioǧlu, R. (2013) Risk factors for chronic kidney disease: an update. Kidney Int Suppl 3: 368–371. 10.1038/kisup.2013.79 PMC4089662 25019021

[B36] Kim, S.M., and Song, I.H. (2020) The clinical impact of gut microbiota in chronic kidney disease. Korean J Intern Med 35: 1305–1316. 32872729 10.3904/kjim.2020.411 PMC7652652

[B37] Lau, W.L., Savoj, J., Nakata, M.B., and Vaziri, N.D. (2018) Altered microbiome in chronic kidney disease: systemic effects of gut-derived uremic toxins. Clin Sci (Lond) 132: 509–522. 29523750 10.1042/CS20171107

[B38] Li, Y., Su, X., Zhang, L., Liu, Y., Shi, M., Lv, C., et al. (2019) Dysbiosis of the gut microbiome is associated with CKD5 and correlated with clinical indices of the disease: a case-controlled study. J Transl Med 17: 228. 31315634 10.1186/s12967-019-1969-1 PMC6637476

[B39] Li, Y., Dai, M., Yan, J., Liu, F., Wang, X., Lin, L., et al. (2021) Colonic dialysis can influence gut flora to protect renal function in patients with pre-dialysis chronic kidney disease. Sci Rep 11: 12773. 34140540 10.1038/s41598-021-91722-1 PMC8211730

[B40] Lu, P.-C., Hsu, C.-N., Lin, I.-C., Lo, M.-H., Yang, M.-Y., and Tain, Y.-L. (2021) The association between changes in plasma short-chain fatty acid concentrations and hypertension in children with chronic kidney disease. Front Pediatr 8: 613641. 33614542 10.3389/fped.2020.613641 PMC7890123

[B41] Lun, H., Yang, W., Zhao, S., Jiang, M., Xu, M., Liu, F., and Wang, Y. (2019) Altered gut microbiota and microbial biomarkers associated with chronic kidney disease. MicrobiologyOpen 8: e00678. 30088332 10.1002/mbo3.678 PMC6460263

[B42] Magliocca, G., Mone, P., Di Iorio, B.R., Heidland, A., and Marzocco, S. (2022) Short-chain fatty acids in chronic kidney disease: focus on inflammation and oxidative stress regulation. Int J Mol Sci 23: 5354. 35628164 10.3390/ijms23105354 PMC9140893

[B43] Magoč, T., and Salzberg, S.L. (2011) FLASH: fast length adjustment of short reads to improve genome assemblies. Bioinformatics 27: 2957–2963. 21903629 10.1093/bioinformatics/btr507 PMC3198573

[B44] McDonough, L.K., Meredith, K.T., Nikagolla, C., Middleton, R.J., Tan, J.K., Ranasinghe, A.V., et al. (2020) The water chemistry and microbiome of household wells in Medawachchiya, Sri Lanka, an area with high prevalence of chronic kidney disease of unknown origin (CKDu). Sci Rep 10: 18295. 33106529 10.1038/s41598-020-75336-7 PMC7589467

[B45] Moffett, J.R., Puthillathu, N., Vengilote, R., Jaworski, D.M., and Namboodiri, A.M. (2020) Acetate revisited: A key biomolecule at the nexus of metabolism, epigenetics, and oncogenesis—Part 2: acetate and ACSS2 in health and disease. Front Physiol 11: 580171. 33304273 10.3389/fphys.2020.580171 PMC7693462

[B46] Mukherjee, A., Lordan, C., Ross, R.P., and Cotter, P.D. (2020) Gut microbes from the phylogenetically diverse genus *Eubacterium* and their various contributions to gut health. Gut Microbes 12: 1802866. 32835590 10.1080/19490976.2020.1802866 PMC7524325

[B47] Neuen, B.L., Chadban, S.J., Demaio, A.R., Johnson, D.W., and Perkovic, V. (2017) Chronic kidney disease and the global NCDs agenda. BMJ Glob Health 2: e000380. 10.1136/bmjgh-2017-000380 PMC5717948 29225940

[B48] Nguyen, H.T., Hongsrichan, N., Intuyod, K., Pinlaor, P., Yingklang, M., Chaidee, A., et al. (2022) Investigation of gut microbiota and short-chain fatty acids in *Strongyloides stercoralis*-infected patients in a rural community. Biosci Microbiota Food Health 41: 121–129. 35854692 10.12938/bmfh.2021-054 PMC9246423

[B49] Olsson, A., Gustavsen, S., Nguyen, T.D., Nyman, M., Langkilde, A.R., Hansen, T.H., et al. (2021) Serum short-chain fatty acids and associations with inflammation in newly diagnosed patients with multiple sclerosis and healthy controls. Front Immunol 12: 661493. 34025661 10.3389/fimmu.2021.661493 PMC8134701

[B50] Pace, F., Carvalho, B.M., Zanotto, T.M., Santos, A., Guadagnini, D., Silva, K.L.C., et al. (2018) Helminth infection in mice improves insulin sensitivity via modulation of gut microbiota and fatty acid metabolism. Pharmacol Res 132: 33–46. 29653264 10.1016/j.phrs.2018.04.008

[B51] Portincasa, P., Bonfrate, L., Vacca, M., De Angelis, M., Farella, I., Lanza, E., et al. (2022) Gut microbiota and short chain fatty acids: implications in glucose homeostasis. Int J Mol Sci 23: 1105. 35163038 10.3390/ijms23031105 PMC8835596

[B52] Ray, K.J., Cotter, S.Y., Arzika, A.M., Kim, J., Boubacar, N., Zhou, Z., et al. (2019) High-throughput sequencing of pooled samples to determine community-level microbiome diversity. Ann Epidemiol 39: 63–68. 31635933 10.1016/j.annepidem.2019.09.002 PMC6996001

[B53] Sabatino, A., Regolisti, G., Brusasco, I., Cabassi, A., Morabito, S., and Fiaccadori, E. (2015) Alterations of intestinal barrier and microbiota in chronic kidney disease. Nephrol Dial Transplant 30: 924–933. 25190600 10.1093/ndt/gfu287

[B54] Sampaio-Maia, B., Simões-Silva, L., Pestana, M., Araujo, R., and Soares-Silva, I.J. (2016) Chapter three—The role of the gut microbiome on chronic kidney disease. In *Advances in Applied Microbiology*. Sariaslani, S., and Gadd, G.M. (eds). Cambridge, MA: Academic Press, pp. 65–94. 10.1016/bs.aambs.2016.06.002 27565581

[B55] Segata, N., Izard, J., Waldron, L., Gevers, D., Miropolsky, L., Garrett, W.S., and Huttenhower, C. (2011) Metagenomic biomarker discovery and explanation. Genome Biol 12: R60. 21702898 10.1186/gb-2011-12-6-r60 PMC3218848

[B56] Shivani, S., Kao, C.Y., Chattopadhyay, A., Chen, J.W., Lai, L.C., Lin, W.H., et al. (2022) Uremic toxin-producing bacteroides species prevail in the gut microbiota of Taiwanese CKD patients: An ana­lysis using the new Taiwan microbiome baseline. Front Cell Infect Microbiol 12: 726256. 35558102 10.3389/fcimb.2022.726256 PMC9086402

[B57] Vaziri, N.D., Wong, J., Pahl, M., Piceno, Y.M., Yuan, J., DeSantis, T.Z., et al. (2013) Chronic kidney disease alters intestinal microbial flora. Kidney Int 83: 308–315. 22992469 10.1038/ki.2012.345

[B58] Vemuri, R., Shankar, E.M., Chieppa, M., Eri, R., and Kavanagh, K. (2020) Beyond just bacteria: Functional biomes in the gut ecosystem including virome, mycobiome, archaeome and helminths. Microorganisms 8: 483. 32231141 10.3390/microorganisms8040483 PMC7232386

[B59] Vlahos, P., Schensul, S.L., Nanayakkara, N., Chandrajith, R., Haider, L., Anand, S., et al. (2019) Kidney progression project (KiPP): Protocol for a longitudinal cohort study of progression in chronic kidney disease of unknown etiology in Sri Lanka. Global Public Health 14: 214–226. 30095037 10.1080/17441692.2018.1508480 PMC6369022

[B60] Wang, Q., Garrity, G.M., Tiedje, J.M., and Cole, J.R. (2007) Naive Bayesian classifier for rapid assignment of rRNA sequences into the new bacterial taxonomy. Appl Environ Microbiol 73: 5261–5267. 17586664 10.1128/AEM.00062-07 PMC1950982

[B61] Wang, S., Lv, D., Jiang, S., Jiang, J., Liang, M., Hou, F., and Chen, Y. (2019) Quantitative reduction in short-chain fatty acids, especially butyrate, contributes to the progression of chronic kidney disease. Clin Sci (Lond) 133: 1857–1870. 31467135 10.1042/CS20190171

[B62] Wang, X., Yang, S., Li, S., Zhao, L., Hao, Y., Qin, J., et al. (2020) Aberrant gut microbiota alters host metabolome and impacts renal failure in humans and rodents. Gut 69: 2131–2142. 32241904 10.1136/gutjnl-2019-319766 PMC7677483

[B63] Weaver, V.M., Fadrowski, J.J., and Jaar, B.G. (2015) Global dimensions of chronic kidney disease of unknown etiology (CKDu): a modern era environmental and/or occupational nephropathy? BMC Nephrol 16: 145. 26282933 10.1186/s12882-015-0105-6 PMC4539684

[B64] Wehedy, E., Shatat, I.F., and Al Khodor, S. (2021) The human microbiome in chronic kidney disease: A double-edged sword. Front Med (Lausanne) 8: 790783. 35111779 10.3389/fmed.2021.790783 PMC8801809

[B65] Wolever, T., Fernandes, J., and Rao, V. (1996) Serum acetate:propionate ratio is related to serum cholesterol in men but not women. J Nutr 126: 2790–2797. 8914950 10.1093/jn/126.11.2790

[B66] Wong, J., Piceno, Y.M., DeSantis, T.Z., Pahl, M., Andersen, G.L., and Vaziri, N.D. (2014) Expansion of urease- and uricase-containing, indole- and p-cresol-forming and contraction of short-chain fatty acid-producing intestinal microbiota in ESRD. Am J Nephrol 39: 230–237. 24643131 10.1159/000360010 PMC4049264

[B67] Xu, X., Nie, S., Ding, H., and Hou, F.F. (2018) Environmental pollution and kidney diseases. Nat Rev Nephrol 14: 313–324. 29479079 10.1038/nrneph.2018.11

[B68] Yao, Y., Cai, X., Fei, W., Ye, Y., Zhao, M., and Zheng, C. (2022) The role of short-chain fatty acids in immunity, inflammation and metabolism. Crit Rev Food Sci Nutr 62: 1–12. 33261516 10.1080/10408398.2020.1854675

[B69] Zeng, H., Umar, S., Rust, B., Lazarova, D., and Bordonaro, M. (2019) Secondary bile acids and short chain fatty acids in the colon: A focus on colonic microbiome, cell proliferation, inflammation, and cancer. Int J Mol Sci 20: 1214. 30862015 10.3390/ijms20051214 PMC6429521

